# Assessment of occupational exposure from radon in the newly formed underground tourist route under Książ castle, Poland

**DOI:** 10.1007/s00411-021-00903-z

**Published:** 2021-03-19

**Authors:** Lidia Fijałkowska-Lichwa, Tadeusz A. Przylibski

**Affiliations:** 1 Faculty of Civil Engineering, Wrocław University of Science and Technology, Wybrzeże S. Wyspiańskiego 27, 50-370 Wrocław, Poland; 2 Faculty of Geoengineering, Mining and Geology, Wrocław University of Science and Technology, Wybrzeże S. Wyspiańskiego 27, 50-370 Wrocław, Poland

**Keywords:** Radon measurements, Radiation protection, Effective dose, Workplace, Construction works, Ventilation

## Abstract

In the present study, ^222^Rn activity concentrations in a newly formed underground tourist route under Książ castle, Poland, were investigated for periods undisturbed and disturbed by construction works. This preliminary assessment is based on the almost 3-year long continuous measurements (28 Oct. 2016–02 Jul. 2019) done with an SRDN-3 instrument. In detail described are radon concentrations for periods of renovation (11 Aug. 2018–10 Oct. 2018), opening (15 Oct. 2018–10 Apr. 2019) and operation and monitoring (11 Apr. 2019–02 Jul. 2019) of the facility. It was observed that after the termination of construction work, when natural ventilation returned to the state preceding this work, the absolute values of radon activity concentration decreased. The mean annual radon concentrations were higher than the reference level of radon concentration in underground spaces recommended by IAEA, ICRP, and by the EU Council Directive for workplaces. They reached 1179 Bq/m^3^ and 943 Bq/m^3^ in 2017 and 2018, respectively. Cyclically recurring daily changes in radon concentrations occurred only in April and October (so-called transitional periods) and only outside the period of construction work. The results confirmed; however, that these changes need not be considered when planning the work in the tunnel. The minimum effective dose rate from radon exposure occurs in colder periods of the year, from November to the end of March, where the mean effective dose rate value was found to be 0.0003 mSv/h. In contrast, the maximum dose rate of 0.014 mSv/h was observed from April to August.

## Introduction

In Poland, like in other countries, the increased ionising radiation in underground spaces is related to the accumulation of radon—a radioactive gas (Chibowski and Komosa 2001; Skowronek et al. 2004; Fijałkowska-Lichwa and Przylibski 2016; Przylibski et al. 2017; Alvarez-Gallego et al. 2015; Bekteshi et al. 2017; Chao et al. 1997; Da Silva et al. 2011; Cucoş Dinu et al. 2017; Dueñas et al. 2011; Dumitru et al. 2015a, b, 2016; Espinosa et al. 2013; Font et al. 2008; Gillmore et al. 2002, 2005; Korhonen et al. 2000; Lario et al. 2006; Liu et al. 2018; Özen et al. 2019; Perrier et al. 2007; Richon et al. 2005; Shahrokhi et al. 2017; Trevisi et al. 2012; Wang et al. 2019; Vaupotič et al. 2001; Zhou et al. 2019). Existing and newly opened underground facilities of various types and functions are becoming workplaces for a growing number of people. These sites are very often restored historic sites (including technological or military heritage sites like underground fortresses, tunnels, shelters, pyramids, tombs, castles, wineries) where underground tourist routes are organized. As a result, more and more people—mainly service staff and guides, sometimes speleologists or researchers—are working in conditions of increased exposure to ionising radiation from radon (mainly from the radioisotope ^222^Rn) and its, also radioactive, progeny (Przylibski and Fijałkowska-Lichwa 2017a, 2017b, 2018a, b, c, d, 2019; Hafez and Hussein 2001; Hafez et al. 2003; Martinez et al. 2005; Vaupotič 2008; Abdelzaher 2011; Youssef and Hanfi 2019; Wysocka 2011; Gruber et al. 2011; Walczak et al. 2017; Ambrosino et al. 2020a, b). In Poland, increasingly underground tourist facilities are being created in the area of Lower Silesia, especially in the Sudetes and the Sudetic Foreland. Owing to their geological structure, these areas are considered so-called radon prone areas. This is related to the occurrence of radium and uranium-rich crystalline rocks lying directly on the surface or at small depths (Strzelecki et al. 1993, 1994; Wołkowicz 2007; Przylibski 2004, 2015, 2018). Formed in the natural uranium-radium radioactive series as a direct product of the alpha decay of ^226^Ra, ^222^Rn, being a noble gas, is released from the rocks in which it has been formed. Released from rocks, ^222^Rn may accumulate in the air of underground engineering structures. The increased level of ^222^Rn activity concentration is partly due to the effective isolation of these structures from the atmosphere and the consequent ineffective ventilation. Moreover, ^222^Rn penetrates inside underground structures from the surrounding rocks due to numerous fractures and fissures in these rocks as well as voids, pores and cracks in the construction materials and structural elements (supports) of the workings. As ^222^Rn accumulates inside a structure, it may reach very high concentrations (Fijałkowska-Lichwa 2014, 2016, 2020; Fijałkowska-Lichwa and Przylibski 2011, 2016, 2020; Olszewski et al. 2005, 2015; Przylibski 1996, 1999, 2015, 2018; Tchorz-Trzeciakiewicz and Solecki 2011; Tchorz-Trzeciakiewicz and Parkitny 2015).

Most countries in Europe and the rest of the world have introduced reference levels for ^222^Rn activity concentrations in public underground facilities, including underground workplaces, based on the recommendations of various specialized international organizations like the International Atomic Energy Agency (IAEA 1996,2003,2014), the International Commission on Radiological Protection (ICRP 1993,1996,2011,2014,2017) and the World Health Organisation (WHO 2009). The reference levels recommended and used in radiological protection were specified as the level of dose or risk or radionuclide concentration above protective (both preventive and corrective) actions should be planned and optimized. Optimization should be applied as appropriate, also below the reference level and not only above (ICRP 2014).

Recently, ICRP revised the upper value for the reference level for radon gas in dwellings from 600 to 300 Bq/m^3^ (ICRP 2011,2014). The effective doses implied by radon concentrations close to this reference level are close to the level of 10 mSv per year for exposure in a dwelling. As an effective dose limit for occupational exposure, ICRP recommended a value of 20 mSv per year averaged over defined 5-year periods (100 mSv in 5 years), with the further provision that the effective dose should not exceed 50 mSv in any single year (ICRP 2011,2014).

For radon gas in dwellings, ICRP recommends the value of 300 Bq/m^3^ as the upper value of the reference level. ICRP also recommends to use the same upper value of 300 Bq/m^3^ as the reference level for radon gas in mixed-use buildings with access for both members of the public and workers, and in workplaces without access for public. However, in any case, national authorities are responsible that the risk due to radiation exposure is kept as low as reasonably achievable, taking societal and economic considerations into account. This includes the control of radiation sources and the establishment of national reference levels (ICRP 2011,2014).

The ICRP recommendations were taken into account by IAEA. The IAEA established a strategy for protection against exposure due to ^222^Rn at workplaces, including an appropriate reference level for ^222^Rn, set at an annual average activity concentration of ^222^Rn of 1000 Bq/m^3^ (IAEA 2014). Regulations for maintaining stability in national standards have been improved and included in the safety standards issued by the IAEA in their regulatory programmes (IAEA 2014).

The World Health Organization reduced the reference level for ^222^Rn and recommended a value of 100 Bq/m^3^. However, the WHO added that if this level cannot be implemented because of specific geological and house construction conditions, the chosen reference level should at least not exceed 300 Bq/m^3^ (WHO 2009).

The European Union has adopted a mandatory reference level of 300 Bq/m^3^ for the mean annual radon concentration at workplaces in its 27 member states (EU Council Directive 2013). It is emphasized that workplaces where the mean annual ^222^Rn activity concentration can be higher than 300 Bq/m^3^ include underground spaces.

In Poland, issues related to ^222^Rn in residential buildings and at work places are regulated by Atomic Law (Law 2000). After the amendment of this law in compliance with EU regulations (EU Council Directive 2013), a reference level of 300 Bq/m^3^ indicated as the average annual radon activity concentration at home and at workplaces was implemented (Law 2000).

The aim of the present work was to address the problem of occupational exposure to ionising radiation in underground engineering structures, including tourist attractions. To this aim, the exposure of workers (mainly service staff and guides, sometimes also speleologists or researchers) to ionising radiation from ^222^Rn and its decay products was assessed in a newly opened underground facility at Książ castle, Poland, being both an example underground workplace and an underground tourist attraction. Changes in ^222^Rn activity concentration in the underground complex were investigated prior to launching the construction works, during these works, and after opening the facility to visitors. Such an approach has not been presented in the literature yet.

Measurements of ^222^Rn at Książ castle are being carried out continuously since 2014. At the beginning, these measurements were carried out in an underground laboratory, while they have been extended since 2016 to the part of the underground, which is being developed for tourists. The results of the present study allowed the establishment of a database on ^222^Rn concentrations at the studied location, which can be used as a supporting tool (main source of information) when the first construction work will be carried out on the site by private owners and local self-government authorities.

## Materials and methods

### Location—general characteristics

The study was conducted in an underground complex under one of the largest castles in Poland. This complex was not accessible to visitors until 15 October 2018. The studied tunnels are part of the Riese complex built by the German paramilitary organization Todt in the Sowie (Owl) Mountains during World War II in 1943–1945. The intended function of these tunnels has not been fully explained yet (Lamparska 1998; Kalarus 2001; Kruszyński 2004; Pawlikowska 2008).

**Fig. 1:**
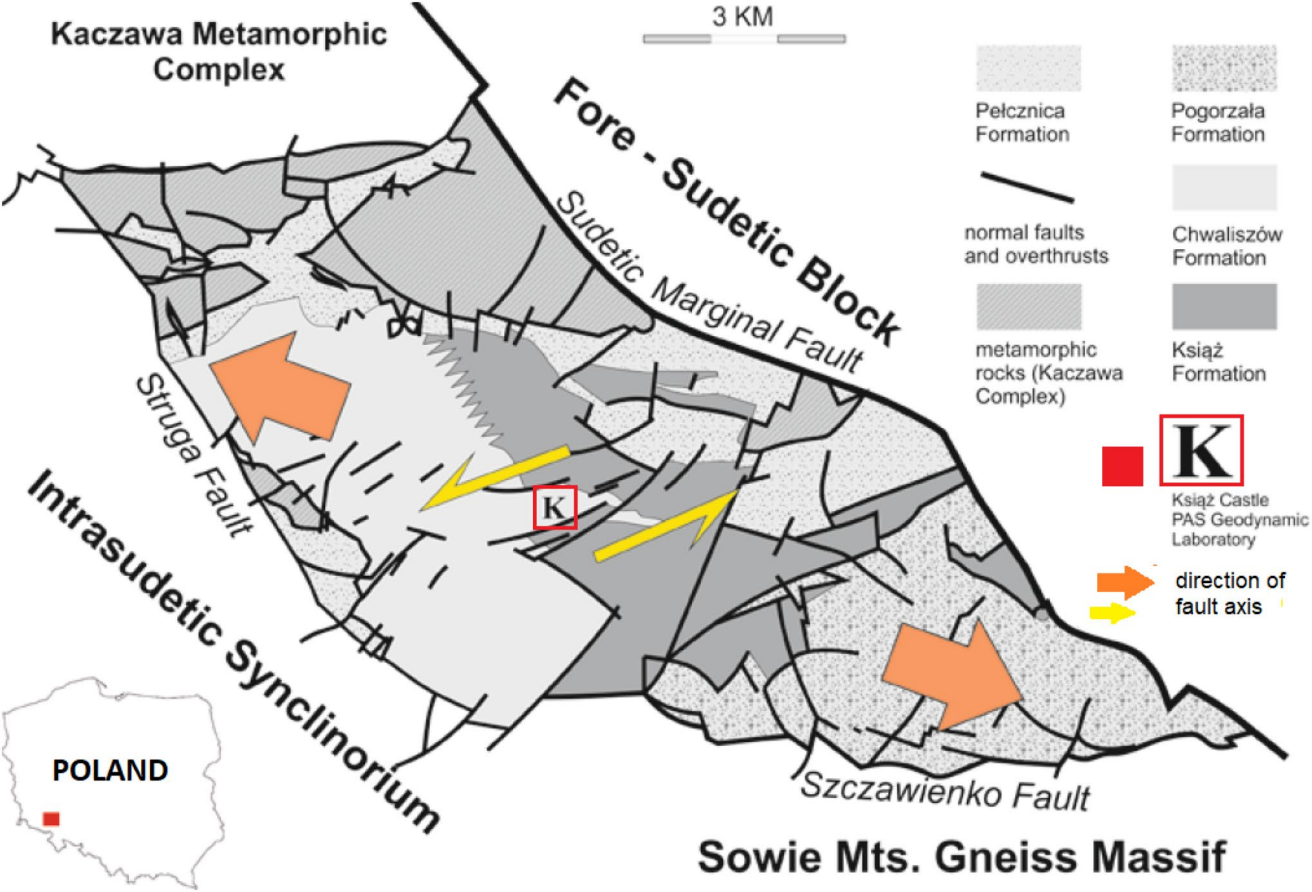
Simplified geological map of Świebodzice Basin with the research site marked as “K” (based on Kaczorowski and Wojewoda 2011); Bottom left corner—location of Świebodzice Basin in Poland

Książ castle is located in the Sudetes, one of the Central European Variscan mountain ranges. The castle is situated within the central part of Świebodzice Basin. This basin is intersected by a dense network of faults. Other large tectonic dislocations form its borders, separating Świebodzice Basin from the neighbouring geological units of the Sudetes. The faults of Struga (in the south), Szczawienko (in the west) and the Sudetic Marginal Fault (in the east) separate Świebodzice Basin from the Intra-Sudetic Basin, the gneiss massif of the Sowie Mountains and the Pre-Sudetic Block respectively. To the north, rocks of the metamorphic Kaczawa Complex are overthrust by the deposits of Świebodzice Basin (Fig. 1; Teisseyre 1956; Porębski 1981; Wojewoda 2016a, b).

Since 1991, the castle has been managed by Zamek Książ partnership in Wałbrzych in collaboration with the Municipality of Wałbrzych. Since 2015, the castle vaults have been used jointly by the Municipality of Wałbrzych and the Polish Academy of Sciences (PAN). According to the bilateral agreement, part of the vaults house the Geodynamic Laboratory of the Space Research Centre (LG CBK). This part represents a separate area excluded from tourist use and set aside for the use of the Polish Academy of Sciences (Kaczorowski 1999a, b, 2009; Kaczorowski et al. 2012, 2018; Kasza et al. 2014, 2018). Since 2014, the Space Research Centre of the Polish Academy of Sciences has been cooperating with Wrocław University of Science and Technology to conduct joint measurements of radon activity concentrations and, in parallel, of any changes in the rock mass kinetics (Fijałkowska-Lichwa nad Przylibski 2016; Przylibski et al. 2020).

The work on the construction of the underground tourist route started on 14 March 2018. The basic work was completed in July 2018 by excavating the staircase shafts and lining them with concrete. In August 2018, adapting the underground complex for tourist use started. At the beginning of October 2018, the first entrance to the object (main entrance) with a staircase measuring 1.2 m^2^ in diameter, located close to entrance no. 2 in the western part of the complex was opened. From August 2018 to mid-October 2018, the system of natural (gravitational) ventilation was adjusted. To increase air flow in the working areas while work was carried out inside the staircase, the doors were opened between 9 a.m. and 6 p.m. The first stage of construction work was completed by the middle of October 2018. The second stage started on 15 October 2018, when the underground tourist route was festively opened to visitors. The last stage of the whole project, i.e.:—operation and monitoring of the route, started on 11 April 2019.

### Ventilation conditions

**Fig. 2:**
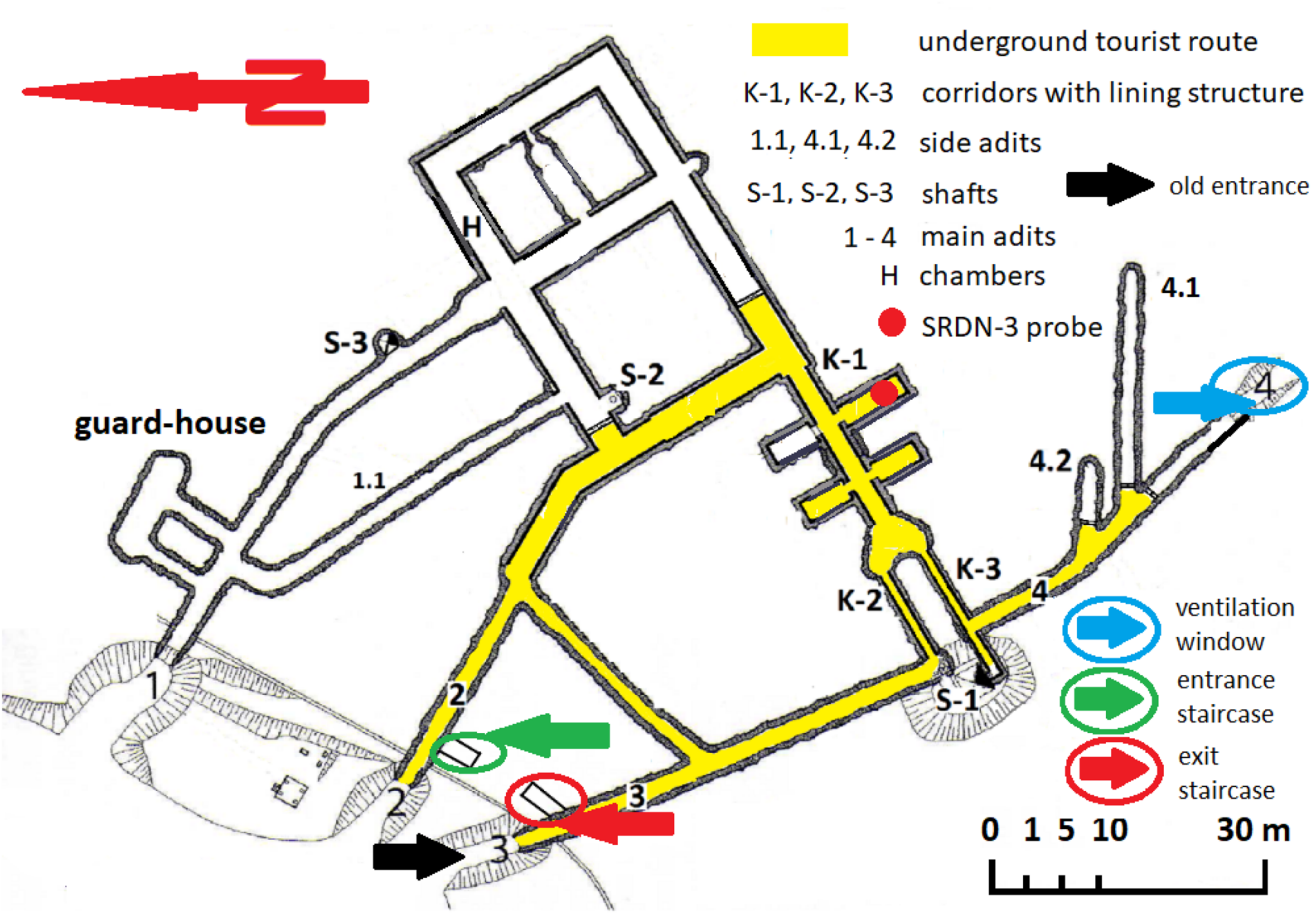
Site plan of the tourist route below the forecourt of Książ castle; red filled circle—location of SRDN-3 probe; figure modified based on the materials provided by facility managers

**Fig. 3:**
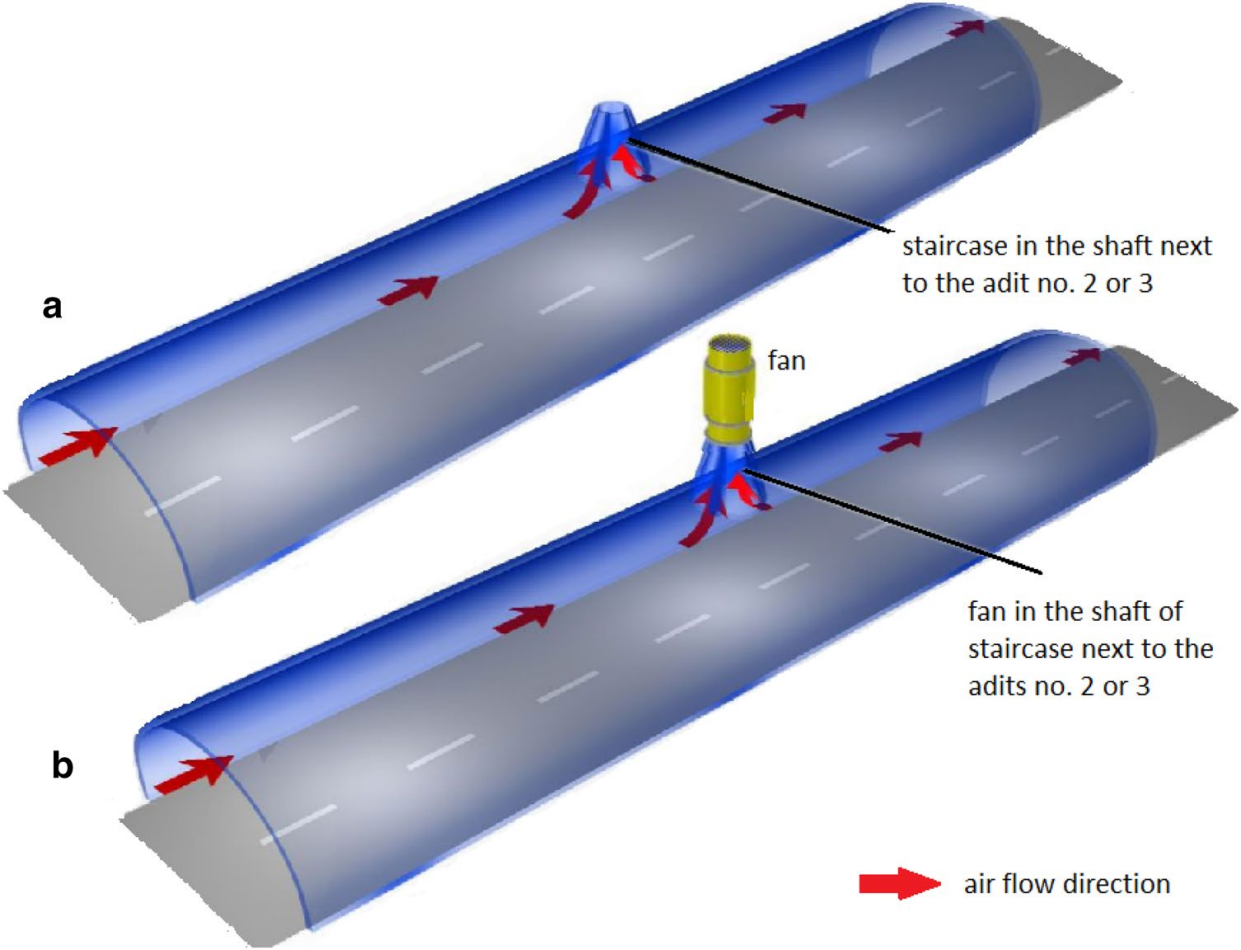
Example plan of natural longitudinal ventilation without fans (**a**) prevalent in winter and mechanical ventilation with a fan in a staircase (**b**) applicable in the underground tourist route in Książ castle (based on Nawrat and Napieraj 2005; Nawrat et al. 2012)

Before the work on the construction of an underground tourist route under Książ castle started, the existing system of underground working places had been relatively well isolated from the atmosphere. Air exchange was possible through natural fractures in the orogen and the door closing the outlet of adit no. 2 (cf. Fig. 2). During the tourist route preparation, the isolation of the space from the atmosphere was partly reduced. For this, two shafts for staircases were excavated near the outlets of adit no. 2 and no. 3, and a ventilation window was opened opposite to the original entrance to and exit—in the partly blocked outlet of adit no. 4. Hence, natural longitudinal ventilation (Fig. 3), without using fans, was employed (Fig. 3a).

Thus, at that time, the research site was not equipped with a mechanical ventilation system (Fig. 3b), and ventilation of the tourist part of the underground complex occurred naturally. Air exchange occurs due to the pressure difference between the inlet (entrance) and the outlet (exit) of the complex—the staircase shafts—and the opened ventilation window in the outlet of adit no. 4 (Fig. 2), and due to any temperature difference between the interior of the complex and the outer atmosphere. The outlet of adit no. 4 is located about 15 m below the door, closing the staircases on the entrance and exit shafts. The temperature inside the working area is almost constant at + 10.2 °C. Thus, the air exchange between the underground complex and the atmosphere is caused by advective and convective air circulation. Natural ventilation conditions improve or deteriorate depending on the prevailing direction of wind. Typically, south-eastern wind pushes the atmospheric air into adit no. 4 (Fig. 2).

Convective air movement is the main mechanism of natural ventilation in the complex from autumn to spring, when distinctly warmer air containing radon is transported from the working areas to the atmosphere. In the transitional periods occurring in spring (April) and autumn (October), when the atmospheric air temperature oscillates around + 10 °C, convective air exchange with the atmosphere is triggered only at night (from the afternoon, through the evening, until the late morning of the following day). In contrast, in summer, the cool air inside the working areas stagnates together with radon accumulating in the spaces of the underground tourist route. Natural ventilation is enhanced by depression resulting from air density differences. This causes a change in the air flow direction in summer as compared to winter. In the warmer season, mainly in summer, the air flows from the north-west to the south, i.e., from the staircase to the outlet of adit no. 4. In winter, air exchange occurs in the opposite direction, and the air is directed from the ventilation window in adit no. 4 to the staircases at the entrance to and exit from the complex (by adit no. 2 and no. 3), i.e., from south to north-west (Fig. 2). The ventilation window in the outlet of adit no. 4 is opened to adjust the ventilation of the working area, especially when a strong wind blows from the south or south-east and forces an atmospheric air stream straight into adit no. 4 (Fig. 2). The air stream that enters underground through the ventilation window in adit no. 4 is directed up the staircase in the shaft next to adits 2 and 3 and then, about 15 m higher is released into the atmosphere (Fig. 2).

### Radon measurements

To monitor ^222^Rn activity concentrations, an SRDN-3 probe—equipped with a semi-conductor detector was used. This device has been used for nearly 10 years in demanding conditions, especially in environments with prolonged or permanent persistence of a relative humidity of 100%. The structure and operating principle of the device is described in detail in Przylibski et al. (2010). The SRDN-3 was calibrated at the Nuclear Physics Institute of the Polish Academy of Sciences in Krakow, Poland, involving certified measuring methods and including various ^222^Rn activity concentrations in a radon chamber. A radon monitor AlphaGUARD PQ2000PRO was used as a reference device (Przylibski et al. 2010).

In terms of the ^222^Rn activity concentration, the detection limit after calibration of the detector was close to 80 Bq/m^3^. The maximum measurable ^222^Rn activity concentration was established by the producer—the Institute of Nuclear Chemistry and Technology (IChTJ) in Warsaw—as 157 MBq/m^3^. This is considerably higher than the values observed so far in underground spaces, i.e., about 50 times higher than concentrations typically observed in the environment (approximately several MBq/m^3^). Typically, measurement uncertainties (mean value ± SE) for concentrations close to the detection limit (80–100 Bq/m^3^) did not exceed 24%, and were between 7 and 12% for ^222^Rn activity concentrations of the order of 500–1000 Bq/m^3^, and less than 5% for ^222^Rn activity concentrations of 5000–10,000 Bq/m^3^ (Przylibski et al. 2010).

Measurements of ^222^Rn activity concentrations were carried out in the working areas lying at the depth of 50 m below the level of the castle forecourt. The underground system is composed of four adits interconnected by numerous corridors and chambers (Fig. 2). To identify the highest ^222^Rn activity concentrations, measurements were performed at the point with the lowest expected ventilation, i.e., the most distant from the entrance to and exit from the underground tourist route (Fig. 2). Because the ^222^Rn concentrations measured at this point represent a worst-case scenario, the corresponding effective doses will probably overestimate somewhat the exposure of workers and tourists.

The SRDN-3 device was placed left of the main gallery fork (K-1), in a concrete-lined chamber which is a part of the main traffic route (Fig. 2). The cross-section of the chamber resembles an ellipse. Its length, measured from the entrance, is about 15 m, the width is about 3.7 m, and the height is about 4.8 m. The chamber is made of reinforced concrete. The SRDN-3 device was placed at a height of about 1 m above the tunnel floor. The registered counts were stored at 1-h intervals and converted to ^222^Rn activity concentrations with the use of a linear calibration equation (Przylibski et al. 2010).

The impact of the reinforced concrete lining structure, which was employed to stabilize the underground structure, on the levels of ^222^Rn activity concentrations recorded inside the working areas of Książ castle was discussed by Fijałkowska-Lichwa (2020). It was shown that the reinforced concrete lining did not affect the character (seasonal, daily) of changes in ^222^Rn activity concentration, neither in the long term (years) nor in the short term (hours or days). Rather, it should be seen as a barrier that reduces the porosity of the rocks and limits the radon flux from the surrounding rocks into the tourist corridors (Fijałkowska-Lichwa 2020).

In the period between 12 January 2017 and 04 April 2017, the measurements were suspended because of a failure of the SRDN-3 detector. The ^222^Rn activity concentrations were taken from a detector operating near the underground tourist route, inside the CBK PAN geodynamic laboratory. These concentrations were included in the present analyses and used for the interpretation of ^222^Rn concentrations in the tourist part of the underground complex at Książ.

### Effective dose calculation

To estimate the mean annual effective dose *E* (mSv/year) from radon and its decay products (^218^Po, ^214^Pb, ^214^Bi and ^214^Po) received by people working in the studied underground workplaces, UNSCEAR guidelines (2000) were employed. Specifically, the effective dose *E* was calculated as a sum of two components: the effective dose resulting from inhalation of ^222^Rn and its daughter products (expressed in mSv) and the effective dose resulting from ^222^Rn intake (also expressed in mSv) (Eq. 1).

1 $$E = C \cdot F \cdot E_{{{\text{iCF}}}}^{ + } C \cdot E_{{{\text{bCF}}}} ,$$

where *C* is ^222^Rn activity concentration in air (Bq/m^3^), and *F* is the radioactive equilibrium factor. The value of *F* was adopted as 0.4—the level proposed for indoor workplaces and tourist caves included in Publication 137 (ICRP 137 2017). Furthermore, *E*_iCF_ is the dose conversion factor for inhalation, with the value of 9.0 (nSv/Bq∙h/m^3^) and *E*_bCF_ is the dose conversion factor for ^222^Rn ingestion and its dissolution in blood, with the value of 1.7 (nSv/Bq∙h/m^3^), adopted from UNSCEAR guidelines (2000,2019).

All effective dose (*E*) values calculated with Eq. 1 are related to 1 h of work or 1 h spent inside the facility. To compare this with the annual effective dose limits set in Appendix 4 to Atomic Law of 29 November 2000 (Law 2000), these values were multiplied with the number of hours an individual worker spent in the working areas of the prepared tourist route in the underground complex of Książ castle in 2018 (Tables 1 and 2). The ICRP proposed also a value of 0.2 as the equilibrium factor *F* for mines (ICRP 137 2017; Annex A, Table A.11). Taking this value instead of the value of 0.4 mentioned above, the effective dose values were calculated with Eq. 1 would be a factor of 2 smaller than those shown in Tables 1 and 2.

**Table 1:** Effective doses from radon intake in 2018 using data on individual working times under the ground

Month	January	February	March	April	May	June	July	August	September	October	November	December	2018
Number of work hours spent under the ground by one guide	23	24	34	59	120	108	121	124	61	53	28	29	784
Maximum effective dose rate (mSv/h)	0.007	0.005	0.005	0.009	0.011	0.012	0.014	0.010	0.006	0.009	0.008	0.008	0.014
Effective dose rate (mSv)	0.2	0.1	0.2	0.5	1.3	1.3	1.7	1.3	0.4	0.5	0.2	0.2	7.8

**Table 2:** Effective doses from intake of radon and its decay products received in selected measurement periods by people spending 1 h in the working areas of the underground tourist route at Książ castle

Measurement period	Number of hourly measurements	Effective dose rates			
		Average ± standard error (mSv/h)	Standard deviation (mSv/h)	Maximum (mSv/h)	Minimum (mSv/h)
01.01.2017–31.12.2017	8675	0.004 ± 0.00002	0.002	0.01	0.0005
01.01.2018–31.12.2018	8754	0.004 ± 0.00001	0.001	0.01	0.0003
**28.10.2016–02.07.2019**	**23,370**	**0.004 ± 0.00001**	**0.002**	**0.01**	**0.0003**
01.01.–31.01.2017	744	0.003 ± 0.00004	0.001	0.006	0.0005
01.01.–31.01.2018	744	0.004 ± 0.00004	0.001	0.008	0.001
01.01.–31.01.2019	744	0.003 ± 0.00003	0.0009	0.006	0.0004
01.02.–28.02.2017	672	0.002 ± 0.00003	0.0007	0.005	0.0006
01.02.–28.02.2018	672	0.004 ± 0.00004	0.001	0.008	0.002
01.02.–28.02.2019	672	0.003 ± 0.00003	0.0009	0.007	0.0004
01.03.–31.03.2017	744	0.003 ± 0.00003	0.0007	0.005	0.0009
01.03.–31.03.2018	744	0.004 ± 0.00004	0.0011	0.007	0.0009
01.03.–31.03.2019	744	0.003 ± 0.00003	0.0009	0.006	0.0003
01.04.–30.04.2017	638	0.005 ± 0.00005	0.001	0.009	0.002
01.04.–30.04.2018	718	0.004 ± 0.00004	0.001	0.007	0.001
01.04.–30.04.2019	720	0.003 ± 0.00003	0.0009	0.006	0.0006
01.05.–31.05.2017	744	0.006 ± 0.00005	0.001	0.011	0.002
01.05.–31.05.2018	744	0.004 ± 0.00004	0.001	0.007	0.0004
01.05.–31.05.2019	744	0.003 ± 0.00004	0.001	0.007	0.0009
01.06.–30.06.2017	720	0.007 ± 0.00005	0.001	0.012	0.003
01.06.–30.06.2018	720	0.004 ± 0.00003	0.0009	0.007	0.001
01.06.–30.06.2019	720	0.004 ± 0.00004	0.001	0.008	0.0009
01.07.–31.07.2017	741	0.008 ± 0.00005	0.001	0.014	0.003
01.07.–31.07.2018	741	0.004 ± 0.000003	0.0009	0.007	0.001
01.08.–31.08.2017	744	0.004 ± 0.00005	0.001	0.010	0.001
01.08.–31.08.2018	744	0.004 ± 0.00003	0.0009	0.006	0.0004
01.09.–30.09.2017	720	0.003 ± 0.00003	0.0008	0.006	0.0007
01.09.–30.09.2018	720	0.003 ± 0.00004	0.001	0.01	0.0006
01.10.–31.10.2016	85	0.005 ± 0.0001	0.001	0.009	0.002
01.10.–31.10.2017	744	0.005 ± 0.0006	0.002	0.009	0.0007
01.10.–31.10.2018	743	0.003 ± 0.00003	0.0009	0.006	0.0003
01.11.–30.11.2016	720	0.004 ± 0.00004	0.001	0.008	0.002
01.11.–30.11.2017	720	0.004 ± 0.00004	0.0009	0.007	0.002
01.11.–30.11.2018	720	0.003 ± 0.00003	0.0008	0.005	0.0007
01.12.–31.12.2016	744	0.004 ± 0.00004	0.001	0.008	0.002
01.12.–31.12.2017	744	0.004 ± 0.00004	0.001	0.007	0.001
01.12.–31.12.2018	744	0.003 ± 0.00003	0.0008	0.005	0.0004

The bold values indicate the entire measurement period

In Poland, the annual effective dose limit for employees is 20 mSv/year (Law 2000, item 1792, app. 4, sections 1.1 and 1.4) and 1 mSv per calendar year for the general public (Law 2000, item 1792, app. 4, section 3).

## Results and discussion

### Seasonal changes in ^222^Rn concentrations

Seasonal variations are well known and characteristic for underground spaces well isolated from the atmosphere (Ambrosino et al. 2019). In Poland, this phenomenon has already been observed in many underground spaces, such as the publicly accessible sites at Kletno, Kowary and Złoty Stok, as well as the CBK PAN Geodynamic Laboratory at Książ (Przylibski 1996, 1999, 2001; Przylibski and Ciężkowski 1999; Fijałkowska-Lichwa 2014, 2016; Fijałkowska-Lichwa and Przylibski 2016, 2020).

**Fig. 4:**
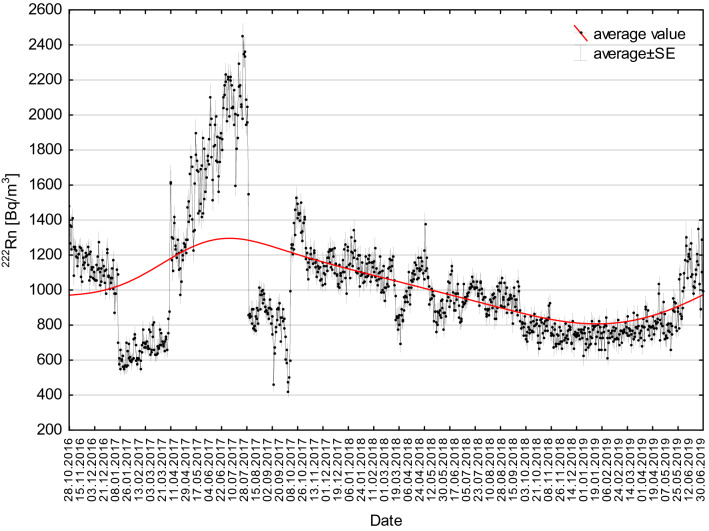
Radon activity concentrations changes as measured throughout the entire observation period from 28 October 2016 to 2 July 2019 at the measurement location (see Fig. 2) below the forecourt of Książ castle. Red line—moving average value of ^222^Rn activity concentration. *SE* standard error

**Fig. 5:**
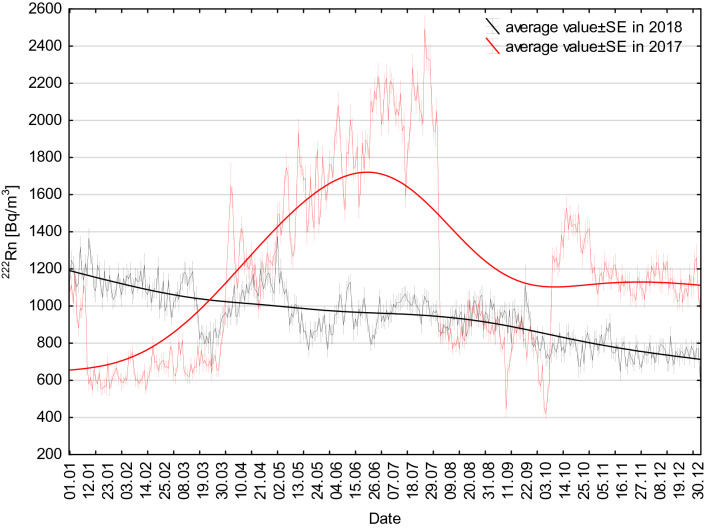
Radon activity concentration changes recorded below the forecourt of Książ castle throughout the 2 years of observation: from 1 January 2017 to 31 December 2017 and from 1 January 2018 to 31 December 2018. Solid line—moving average value of ^222^Rn activity concentration. *SE* standard error

**Fig. 6:**
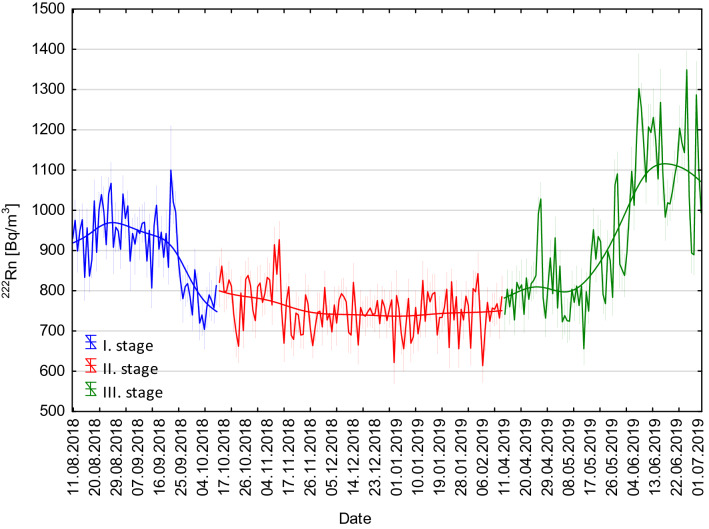
Courses of changes in ^222^Rn activity concentration registered at successive stages of the facility creation: stage 1—adaptation works from 11 August 2018 to 10 October 2018, stage 2—opening of the tourist route from 15 October 2018 to 10 April 2019, stage 3—facility operation and monitoring of the ionising radiation exposure conditions from 11 April 2019 to 02 July 2019. Solid line—moving average value of ^222^Rn activity concentration. Mean ± SE; *SE* standard error

In the context of the present study, seasonal changes in ^222^Rn activity concentration occurred throughout all measurement periods, but their course was varied in years disturbed (2018) and undisturbed (2017) by construction works, respectively (Figs. 4 and 5). The analyses performed as part of the present study also took into account all stages of the study route creation (Fig. 6).

**Fig. 7:**
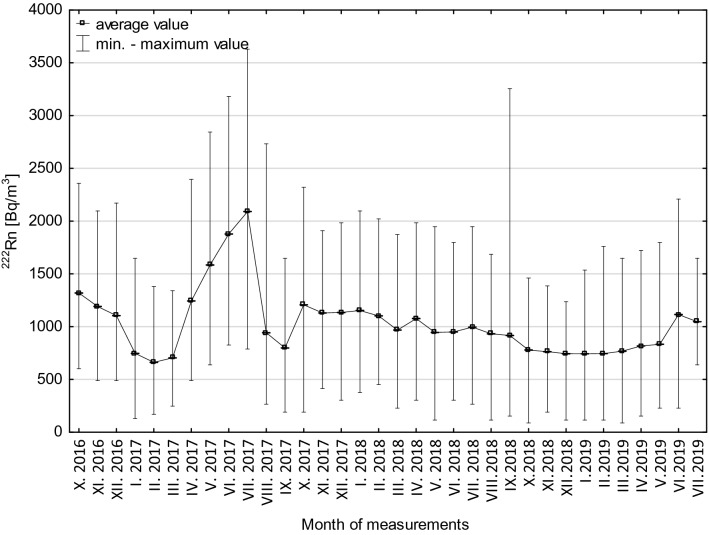
Box-and-whisker plot of measured ^222^Rn activity concentrations registered in a particular month between 28 October 2016 and 2 July 2019

In 2018, the ^222^Rn activity concentrations decreased slowly but steadily throughout the year, from 1200 to 700 Bq/m^3^ (Fig. 4). The amplitudes of seasonal changes were comparable throughout the year 2018 and fell within the range of 100–300 Bq/m^3^ (Fig. 4). This was due to the ongoing construction works resulting in an increased ventilation of the complex. At the first stage of tourist route creation (11 August 2018 to 14 October 2018), the course of changes in ^222^Rn activity concentration was largely influenced by a change in the intensity and volume of the air stream flowing through the underground complex. This was related to typical seasonal changes, additionally enhanced by increased ventilation intensity caused by the opening of staircases near adit no. 2 and no. 3 during the facility working hours (9:00–18:00) (Figs. 4, 5, 6). At the second stage (15 October 2018 to 10 April 2019), the registered mean values of ^222^Rn activity concentration remained at a practically stable level of 750 Bq/m^3^. They also showed small yet irregular changes of 50–100 Bq/m^3^ above or below the mean value. Such a course of change was significantly affected by stable (fixed) conditions of intense airing (natural convective ventilation) of the facility (Fig. 6). At the last stage (11 April 2019 to 2 July 2019) ^222^Rn activity concentrations showed irregular yet large fluctuations characteristic of the so-called transitional period (Fig. 6). The amplitude of these changed ranged from 50 to 200 Bq/m^3^ in relation to the mean value (Fig. 6). Between May and July 2019, the concentrations increased from the average level of 800 to slightly more than 1100 Bq/m^3^ (Figs. 6 and 7).

In 2017, undisturbed by construction works, the changes in ^222^Rn activity concentrations looked different. Low values were recorded between January and March (600–800 Bq/m^3^), in the exceptionally cold months of August and September (400–1000 Bq/m^3^) and in November and December (about 1100 Bq/m^3^). The highest mean values, exceeding 2200 Bq/m^3^, were recorded in summer 2017 (Figs. 5 and 7, Table 3). The results obtained demonstrate that even in February 2017, where the lowest radon activity concentrations were measured (average 662 Bq/m^3^), the mean ^222^Rn activity concentration was more than twice as high as the reference level recommended for workplaces by the European Commission (EU Council Directive 2013). The mean annual ^222^Rn activity concentration was slightly higher (1179 Bq/m^3^) in 2017 and lower (943 Bq/m^3^) in 2018, because of the ongoing construction works (Table 3).

**Table 3:** ^222^Rn activity concentrations recorded throughout the observation period between 28 October 2016 and 2 July 2019 in the working areas of the underground tourist route at Książ castle

Measurement period	Number of hourly measurements	Average (Bq/m^3^)	Median (Bq/m^3^)	Minimum (Bq/m^3^)	Maximum (Bq/m^3^)	Standard deviation (Bq/m^3^)	Standard error (Bq/m^3^)
01.01.–31.12.2017	8675	1179	1087	130	3629	538	5.8
01.01.–31.12.2018	8754	943	938	88	3255	280	3.0
**28.10.2016–02.07.2019**	**23,370**	**1025**	**938**	**88**	**3629**	**419**	**2.7**
01.01.–31.01.2017	744	747	714	130	1648	292	10.7
01.01.–31.01.2018	744	1153	1125	377	2096	264	9.7
01.01.–31.01.2019	744	743	751	116	1536	228	8.3
01.02.–28.02.2017	672	662	637	169	1379	185	7.1
01.02.–28.02.2018	672	1102	1087	452	2021	259	10.0
01.02.–28.02.2019	672	745	751	116	1760	232	9.0
01.03.–31.03.2017	744	704	676	247	1340	186	6.8
01.03.–31.03.2018	744	968	975	228	1872	280	10.3
01.03.–31.03.2019	744	767	751	88	1648	226	8.3
01.04.–30.04.2017	638	1243	1237	489	2395	301	11.9
01.04.–30.04.2018	718	1077	1087	302	1984	267	10.0
01.04.–30.04.2019	720	817	788	153	1723	234	8.7
01.05.–31.05.2017	744	1584	1573	639	2844	338	12.4
01.05.–31.05.2018	744	945	938	116	1947	270	9.9
01.05.–31.05.2019	744	833	826	228	1797	254	9.3
01.06.–30.06.2017	720	1878	1872	826	3180	359	13.4
01.06.–30.06.2018	720	950	938	302	1797	244	9.1
01.06.–30.06.2019	720	1115	1087	228	2209	318	11.8
01.07.–31.07.2017	741	2091	2059	788	3629	369	13.6
01.07.–31.07.2018	741	996	975	265	1947	236	8.7
01.08.–31.08.2017	744	942	863	265	2732	324	11.9
01.08.–31.08.2018	744	932	938	116	1685	247	9.0
01.09.–20.09.2017	720	798	788	190	1648	221	8.3
01.09.–30.09.2018	720	917	900	153	3255	267	10.0
28.10.–31.10.2016	85	1319	1349	601	2358	314	34.1
01.10.–31.10.2017	744	1207	1274	190	2321	415	15.2
01.10.–31.10.2018	743	779	751	88	1461	234	8.6
01.11.–30.11.2016	720	1191	1199	489	2096	266	9.9
01.11.–30.11.2017	720	1130	1125	415	1910	251	9.4
01.11.–30.11.2018	720	763	751	190	1386	213	7.9
01.12.–31.12.2016	744	1105	1106	489	2171	259	9.5
01.12.–31.12.2017	744	1134	1125	302	1984	259	9.5
01.12.–31.12.2018	744	744	751	116	1237	210	7.7

The bold values indicate the entire measurement period

### Daily changes in ^222^Rn concentrations

**Fig. 8:**
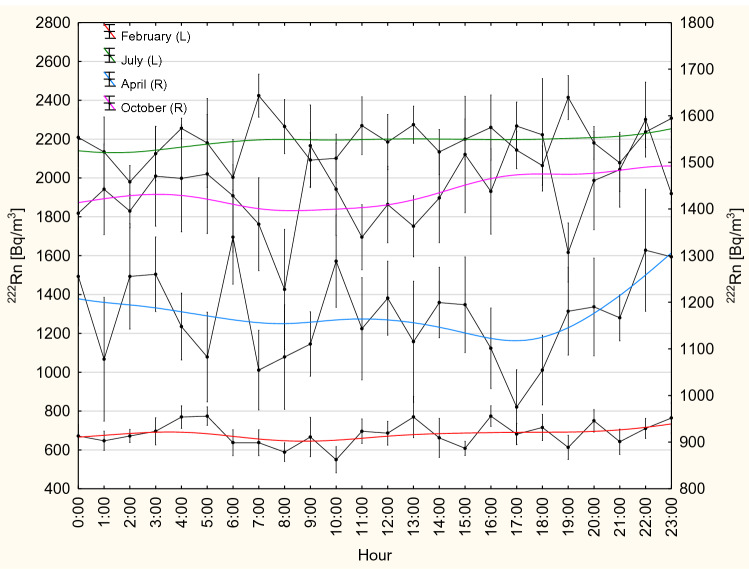
Courses of hourly changes in ^222^Rn activity concentration registered on 7 consecutive days in winter (l3–20 February 2017), in spring (l3–20 April 2017), in summer (13–20 July 2017) and in autumn (13–20 October 2017), during a period undisturbed by construction works. Solid lines: moving average fit; R—right *y* axis; L—left *y* axis;  mean ± SE; *SE* standard error

For the period undisturbed by construction works, hourly changes in ^222^Rn activity concentration were analyzed by studying 7-day periods in April, July, October and February, representing the four seasons of 2017 (spring, summer, autumn and winter), respectively (Fig. 8). Given the observed fluctuations, no significant daily changes in ^222^Rn activity concentration can be deduced. In fact, the identified concentrations differed by no more than 10% from the corresponding mean values (Fig. 8). In February and July, a similar situation was observed. In both cases, there is a rather stable natural ventilation, which is efficient in winter and very inefficient in summer. As a consequence, ^222^Rn activity concentrations differed only about 100 Bq/m^3^ and 200 Bq/m^3^ from their mean values of about 700 Bq/m^3^ and around 2200 Bq/m^3^, respectively (Fig. 8).

In the so-called transitional periods in April and October, the observed daily changes in ^222^Rn concentrations were characterized by unstable and changing conditions of natural ventilation (Fig. 8). In April, higher values than in the morning and around noon were registered in the afternoon and at night (Fig. 8). In October, lower values were recorded until noon (12:00) as compared to the afternoon and at night, where ^222^Rn activity concentrations increased. The mean values of ^222^Rn activity concentration were slightly higher in October than in April, with values in the range 1400–1500 Bq/m^3^ (Fig. 8). In April, the mean ^222^Rn activity concentrations fell within the range of 1100–1300 Bq/m^3^ (Fig. 8). The results shown in Fig. 8 suggest that only in April and October daily changes in ^222^Rn concentrations may be relevant for choosing hours of work (including the opening hours for visitors).

### Dose assessment

Since January 2018, workers (mainly tour guides, maintenance workers and researchers) have been spending an average of 8 h a day inside the facility, including 80% of the time spent inside the underground workings and the remaining 20% outside. For individual workers, the work time schedule was established based on the personal interviews with the facility managers and the work time register for the guides (Table 1).

It was estimated that the lowest average effective dose from ^222^Rn and its decay products was received by employees in February 2017 and the highest in July 2017. The mean effective dose rates for 2017 and 2018 and for the whole measurement period, were 0.004 mSv/h (Table 2). The maximum effective dose rate of 0.014 mSv/h was calculated for July 2017, while the minimum effective dose rate—0.0004 mSv/h—was calculated for May and August 2018. However, after the recommended installation of a mechanical (forced) ventilation system, the observed changes in radon activity concentrations should be mitigated.

## Conclusions

The present assessment of exposure to ionising radiation due to radon inhalation of workers employed in the underground complex of Książ castle and potential future visitors, was based on continuous radon monitoring, performed before, during and after construction works in the study area. Owing to the measured average annual ^222^Rn activity concentrations, the corresponding effective doses of the employees (considering working time, exposure duration and radiation exposure) were estimated. It was shown that the average annual ^222^Rn activity concentration in the air of underground spaces at Książ castle, exceeded the reference level of 300 Bq/m^3^ recommended by the European Commission. Specifically, in 2017, this reference level was exceeded almost fourfold, and in 2018 over threefold, reaching 1179 Bq/m^3^ and 943 Bq/m^3^, respectively. In the whole almost 3-year-long research period, the mean radon concentration was 1,025 Bq/m^3^. The highest radon concentrations were recorded in the spring and summer months, i.e., from April to July. In July 2017, a maximum radon concentration of 3629 Bq/m^3^ was recorded. It should be emphasized that opening (in 2018) new staircases for tourists in shafts and opening a ventilation window in one of the adits have improved the ventilation in the studied underground area. As a result, ^222^Rn activity concentrations decreased, but are still higher than the recommended reference level of 300 Bq/m^3^.

In the period undisturbed by construction works, ^222^Rn activity concentrations showed some seasonal variations characteristic of underground spaces well isolated from the atmosphere. After launching the adaptation work in August 2018, the character of the observed changes in ^222^Rn activity concentration was influenced by the forced ventilation conditions in the workings, i.e., the size of the flowing air stream and the direction of its circulation. As a result, the level of ^222^Rn activity concentration measured in the warmer season (May–September) was similar or slightly lower than that in autumn and winter. The direction of air flow, mainly in the summer, indicated circulation from the north-west to the south. The air was released from the working areas to the atmosphere through a ventilation window located in adit no. 4, about 15 m below the level of the staircase. In winter, air exchange occurred in the opposite direction. The ventilation conditions in the facility affected the distribution of daily changes in ^222^Rn activity concentration only in the so-called transitional periods (April–October), where they were also undisturbed by construction work. Relevant and predictable daily changes in radon concentration occurred in no more than about a dozen days a year—a few days in spring and a few in autumn. Because of this and because significant differences did not exceed 10% from the corresponding mean values, daily changes in ^222^Rn activity concentration are not considered important in the planning of working time under the ground.

The estimated average effective dose rates (effective dose per 1 h of work in the underground spaces under Książ castle) varied from 0.003 to 0.005 mSv/h. In general, for members of the public the mean annual effective dose should not significantly exceed 1 mSv/year. The present study showed that members of the general public, chiefly tourists, but also underage persons like pupils or apprentices spending no more than 1 h a day once a year in the studied facility (note that the duration of a touristic tour is about 40 min), will not receive an effective dose higher than 1 mSv/year. For a worker, the annual effective dose could reach, at worst, 7.8 mSv within a year. Although this value is lower than the limit of effective dose for employees (20 mSv/year), it means that in the present conditions, the employees could be exposed to ionising radiation exceeding 6 mSv/year and 1 mSv/year at best. However, it must be noted that the measurement location was selected in such a way that the ^222^Rn activity concentration measured was highest for the whole underground complex. Based on the results obtained, it is recommended to base environmental dosimetry in the studied underground facility on radon concentration monitoring. Of course, routine radon monitoring should be based on the measurements at several locations along the tourist route. To select the best method (individual or environmental) of monitoring, the expected effective dose and time of exposure should be taken into account.

The occupational exposure to ionising radiation from radon and its progeny in the underground tourist route at Książ castle is a vital problem and it requires a solution compliant with the Polish regulations of the amended Atomic Law and guidelines appended to this law (Law 2000, Item 1792 with appendices). One method of improving working conditions there is increasing the effectiveness of ventilation in working areas. The whole tourist route should be supplied with atmospheric air, not only during time periods with limited natural air exchange (mostly during the transitional periods), but also during the so-called tourist season in summer, when many tourists and service personnel are present. One proposed way is the application of a natural longitudinal ventilation system with fans (Fig. 3b). In such a system, air circulation is triggered by overpressure generated by fans placed near the entrance portal (in adit no. 2) or near the exit of the facility (in adit no. 3). Also worth considering is the installation of a radon monitoring system, which would provide continuous (online) information on the radiation situation along the tourist route. Such a system should include detectors installed at several (3–5) measurement locations. Furthermore, it is necessary to record the working time (exposure time) of any workers in the underground.

The results of the present study demonstrate the importance of radon monitoring in underground facilities, as a model of good practice for radiological protection of workers. Such kind of monitoring should be made mandatory by international and national authorities, for all facilities in similar locations in Poland and worldwide.

## Data Availability

All relevant data and materials are presented in the paper.
